# Depth-dependent Metagenome-Assembled Genomes of Agricultural Soils under Managed Aquifer Recharge

**DOI:** 10.1038/s41597-025-05218-y

**Published:** 2025-05-24

**Authors:** Júlia Brandão Gontijo, Laibin Huang, Elad Levintal, Cristina Prieto García, Christian B. Erikson, Adolfo Coyotl, William R. Horwath, Helen E. Dahlke, Jorge L. Mazza Rodrigues

**Affiliations:** 1 Department of Land, Air, and Water Resources, University of California, Davis, CA 95616 USA; 2 Department of Biology, Saint Louis University, St. Louis, MO 63103 USA; 3 Zuckerberg Institute for Water Research, The Jacob Blaustein Institutes for Desert Research, Ben-Gurion University of the Negev, Sde Boker Campus, 84990 Be’er Sheva, Israel; 4 Environmental Genomics and Systems Biology Division, Lawrence Berkeley National Laboratory, Berkeley, CA 94720 USA

**Keywords:** Metagenomics, Environmental sciences

## Abstract

Managed Aquifer Recharge (MAR) systems, which intentionally replenish groundwater aquifers with excess water, are critical for addressing water scarcity exacerbated by demographic shifts and climate variability. To date, little is known about the functional diversity of the soil microbiome at different soil depth inhabiting agricultural soils used for MAR. Knowing the functional diversity is pivotal in regulating nutrient cycling and maintaining soil health. Metagenomics, particularly Metagenome-Assembled Genomes (MAGs), provide a powerful tool to explore the diversity of uncultivated soil microbes, facilitating in-depth investigations into microbial functions. In a field experiment conducted in a California vineyard, we sequenced soil DNA before and after water application of MAR. Through this process, we assembled 146 medium and 14 high-quality MAGs, uncovering a wide array of archaeal and bacterial taxa across different soil depths. These findings advance our understanding of the microbial ecology and functional diversity of soils used for MAR, contributing to the development of more informed and sustainable land management strategies.

## Background & Summary

Managed Aquifer Recharge (MAR) systems stand at the forefront of strategies to safeguard groundwater resources, especially in regions grappling with escalating water groundwater overdraft intensified by demographic shifts and climate variability^1^. MAR systems play a pivotal role in replenishing aquifers and maintaining their sustainability amid mounting challenges. Surface spreading MAR methods such as agricultural managed aquifer recharge (Ag-MAR), where farm fields, orchards or vineyards are flooded with excess water to replenish groundwater^2^, has been associated with different environmental risks, including the leaching of nitrogen (N), mainly in nitrate (${{\rm{NO}}}_{3}^{-}$) form, and labile carbon (C) as reported in previous studies^2–4^. The soil microbial communities inhabiting Ag-MAR sites are instrumental in mediating these key processes, underlining their significance in mitigating these environmental risks^3–5^.

In recent years, the advent of high-throughput sequencing has revolutionized microbial ecology, allowing scientists to determine the whole microbial community circumventing the need for microbial culturing and enabling the discovery of numerous unidentified taxa and their novel functions^6^. While the 16S rRNA gene amplicon sequencing has significantly expanded our understanding of microbial diversity in Ag-MAR systems, its limitations in resolving closely related species and their functional capacity underscore the indispensability of metagenomics^4^. Furthermore, the reconstruction of microbial genomes from metagenome-assembled genomes (MAGs) has been proposed as a possible solution to access not-yet-cultivated microbial species^7,8^. Hence, MAGs hold an immense potential for unraveling the versatile microbial communities in their novelty associated with phylogeny, metabolism, and physiology in response to Ag-MAR application.

As one of the first, Huang *et al*.^4^ assessed microbial communities and N and C cycling dynamics in a table grape vineyard characterized by a fine sandy loam (58–81% sand, 4–9% clay, and 14–32% silt^2^) in response to Ag-MAR. This study shed light on how soil microbiomes respond to rewetting events, particularly the microbial processes involved in N transformations. Therefore, analyzing MAGs from soil samples under Ag-MAR systems provides a unique opportunity to explore microbial functional capacities related to nutrient and carbon cycles in these environments. Furthermore, understanding soil microbiome responses to Ag-MAR practices informs strategies for optimizing agricultural water management and promoting sustainable land use practices, crucial for the long-term health of groundwater reservoirs and agricultural ecosystems.

**Fig. 1:**
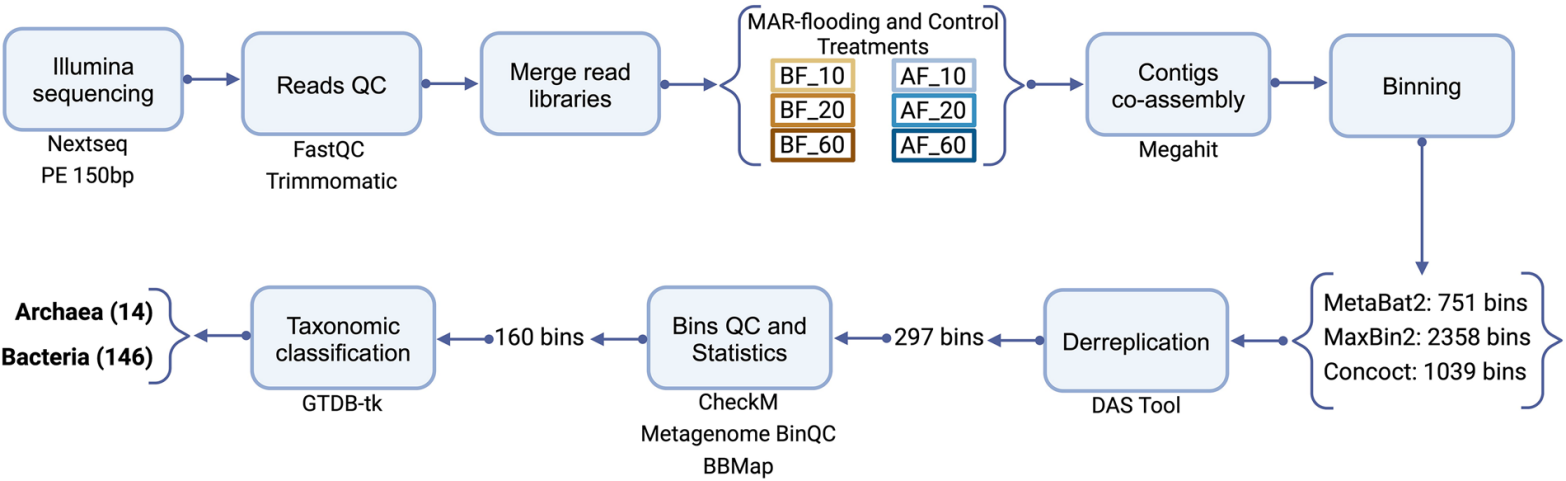
Workflow used for assembling genomes from metagenomes in this study.

Building on the Ag-MAR study conducted by Levintal *et al*.^2^ and Huang *et al*.^4^ in 2021 in a vineyard (*Vitis vinifera* L.) in Fresno, California, we decided to sequence the DNA extracted from field soil samples collected before and after flooding using a high throughput metagenomic approach. Genome assemblies from the metagenomic sequencing data and functional potential analysis were performed on the KBase 2.1.9 platform^9^. Figure 1 summarizes the analytical workflow followed in this study. Three replicates of metagenomic sequencing were used for soil samples obtained from three depths (10, 20, 60 cm) and each treatment (e.g. MAR-flooding and their respective controls): before flooding - 10 cm (BF_10), before flooding - 20 cm (BF_20), before flooding - 60 cm (BF_60), after flooding - 10 cm (AF_10), after flooding - 20 cm (AF_20), and after flooding - 60 cm (AF_60), totaling 36 metagenomes. The results obtained from the read filtering and assembly steps are available on Table 1.

**Table 1:** Summary of reads filtering and assembly results.

Co-assembly	Sample	Paired-end raw reads	Paired-end filtered reads	Merged reads library	Contigs >2000 bp	N50	L50	MAGs
BF_10	1P11	136,227,282	102,692,410	309,038,230	204,730	3,942	53,229	17
	1P21	146,124,630	109,670,274					
	1P31	126,531,622	96,675,546					
BF_20	1P12	125,180,866	94,593,378	301,541,082	206,686	4,225	50,058	12
	1P22	137,312,776	105,092,632					
	1P32	132,748,132	101,855,072					
BF_60	1P13	181,405,362	136,340,206	349,148,074	287,486	4,380	69,629	23
	1P23	162,431,912	123,792,382					
	1P33	115,509,294	89,015,486					
AF_10	2P11	127,969,194	95,420,604	269,246,124	160,718	3,842	41,928	11
	2P21	127,330,848	95,193,474					
	2P31	103,245,918	78,632,046					
AF_20	2P12	125,837,256	95,269,868	292,384,044	166,722	4,010	428,55	12
	2P22	135,058,382	102,008,240					
	2P32	125,466,112	95,105,936					
AF_60	2P13	102,173,922	75,190,368	258,932,248	157,008	4,422	36,683	20
	2P23	122,811,568	92,586,466					
	2P33	117,782,694	91,155,414					
Control_BF_10	1P41	112,394,634	84,909,334	255,733,194	126,206	3,493	35,807	8
	1P11A1	122,255,814	91,651,878					
	1P11B1	104,163,870	79,171,982					
Control_BF_20	1P42	97,461,072	74,253,316	245,714,892	139,318	3,878	35,552	10
	1P11A2	128,036,342	97,455,530					
	1P11B2	98,762,482	74,006,046					
Control_BF_60	1P43	126,607,590	95,213,806	285,037,508	204,635	4,406	48,416	14
	1P11A3	134,363,164	99,378,470					
	1P11B3	119,389,420	90,445,232					
Control_AF_10	2P41	102,565,886	76,491,926	231,578,874	109,286	3,514	29,982	10
	2P11A1	112,764,340	84,832,264					
	2P11B1	92,177,184	70,254,684					
Control_AF_20	2P42	99,732,500	75,081,978	231,490,272	110,917	3,947	28,104	12
	2P11A2	111,884,474	84,304,648					
	2P11B2	94,104,484	72,103,646					
Control_AF_60	2P43	111,663,518	84,510,488	234,178,732	127,184	4,380	30,315	11
	2P11A3	101,981,680	77,542,758					
	2P11B3	95,923,134	72,125,486					

**Fig. 2:**
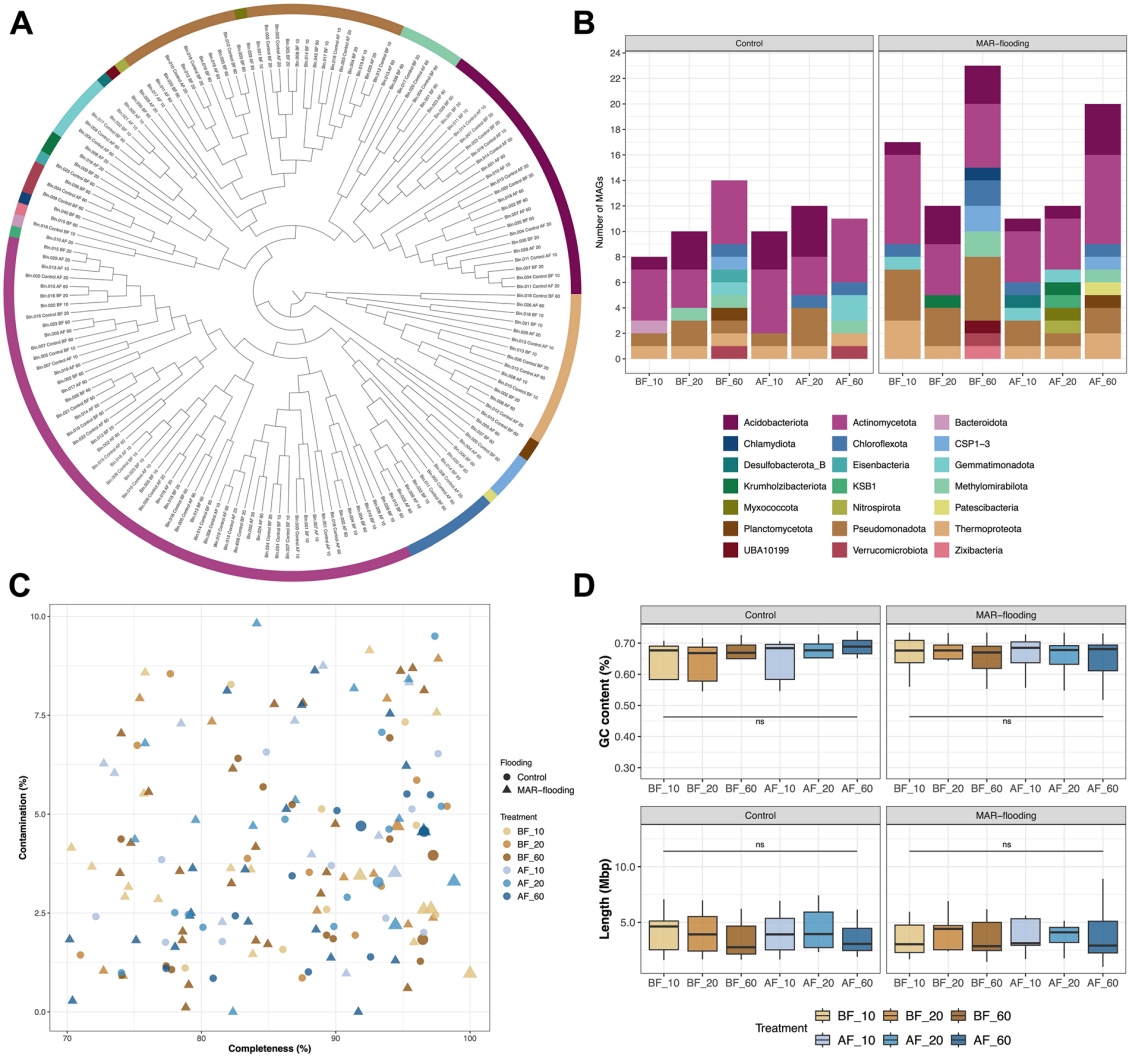
(**A**) Phylogenetic tree of the MAGs using a set of 49 core, universal genes as defined by COG gene families^9^. (**B**) The distribution of all MAGs at the phylum level. (**C**) The relationship between completeness, contamination, and MIMAG standards (Bowers *et al*.^11^), with high-quality MAGs highlighted by larger shapes. (**D**) Boxplots comparing the distribution of GC content (%) and genomic size (Mbp) of the MAGs among the treatments. No statistically significant differences (ns; p < 0.05) were observed in completeness, contamination, GC content, or genome length across treatments and depths, as determined by the Kruskal-Wallis test.

We recovered 14 archaeal (phylum Thermoproteota, class Nitrososphaeria) and 146 bacterial (phyla Acidobacteriota (24), Actinomycetota (56), Bacteroidota (1), Chlamydiota (1), Chloroflexota (8), CSP1-3 (4), Desulfobacterota_B (1), Eisenbacteria (1), Gemmatimonadota (6), Krumholzibacteriota (2), KSB1 (1), Methylomirabilota (6), Myxococcota (1), Nitrospirota (1), Patescibacteria (1), Planctomycetota (2), Pseudomonadota (25), UBA10199 (1), Verrucomicrobiota (3) and Zixibacteria (1)) MAGs. The diversity and MAG-derived results varied among different treatments (Fig. 2, **Quality Metrics File**^10^). No bins could be classified at the species level. Additionally, 53 MAGs could not be assigned at the genus level, 11 at the family level and two at order level, highlighting the potential of this approach to reveal the yet-unknown microbial diversity of Ag-MAR soils.

Following the Minimum Information about a Metagenome-Assembled Genome (MIMAG) parameters defined by Bowers *et al*.^11^, we considered as high-quality MAGs only the assemblies that presented ≥90% of completeness, ≤5% of contamination, at least one copy of the 5S, 16S and 23S rRNA genes and ≥18 tRNAs. The MAGs that did not meet all these criteria and showed completeness >70% and contamination <10% were considered as medium-quality assemblies. Therefore, our analysis generated in total 146 medium and 14 high-quality MAGs (Fig. 2C), with GC% varying from 29 to 74% and genomic size from varying 1.0 to 13.2 Mbp (Fig. 2D). No statistically significant differences (p < 0.05) were observed in completeness, contamination, GC content, or genome length across the different treatments and depths.

Our findings significantly enhance knowledge of microbial communities at different depths in agricultural systems undergoing Ag-MAR. Additionally, the novelty analysis of these MAGs may shed light on the novel functional capabilities of the underrepresented or uncultured microbes, thereby enhancing our comprehension of their ecological roles across various soil depths. In light of the connections between these genomes and critical biogeochemical cycles, our results serve as a valuable resource for further investigations into the functional responses of the soil microbiome concerning agricultural practices, soil health and climate change.

## Methods

### Site location and sampling

The flooding experiment for Ag-MAR took place in a Thompson seedless grape vineyard (*Vitis vinifera* L.) located at Kearney Agricultural Research and Extension Center in Fresno, California (36°36′02.9″N 119°30′39.2″W). Detailed information about this experiment can be found in previous studies^2,4^. In summary, we divided the whole vineyard into six individual subplots to test two treatments with three replicates. Three subplots were flooded for Ag-MAR and three sub-plots served as controls only receiving natural precipitation. The vineyard was flooded for 4 weeks with an infiltration rate of ∼0.088 ± 0.031 m/day, using groundwater as the water source. We collected a total of 36 soil samples (18 from control plots and 18 from flooded plots) using a core sampler (diameter: 10 cm) at three soil depths (10, 20, 60 cm) before and 4 weeks after flooding. The samples were stored in −80 °C for DNA sequence analyses.

### Metagenome assembly and binning

DNA was extracted from these soil samples using the DNeasy Powersoil kits following the manufacturer’s instructions (Qiagen, CA, USA) and sequenced using the NovaSeq Illumina platform with 151 bp paired-end reads in the DNA Technologies Sequencing Core of the Genome Center at UC Davis. A total of 4,317,379,358 paired-end raw reads were retrieved with an average of 119,927,204 for each sample.

The assembly of genomes from the metagenomes was performed on the KBase plataform^9^ and the parameters used for each step are available in the Table 2. Initially, raw metagenomic sequences were filtered and trimmed using Trimmomatic 0.36^12^. This process comprises removing sequencing adapters, and sequences with lengths lower than 70 bp and Phred score lower than 30. The quality of the sequences was validated using FastQC 0.11.5^13^. After quality control, the three replicates of each treatment were merged into a single sequence library each using the app Merge Reads Libraries 1.0.1^9^. Co-assembly was performed with MEGAHIT 1.2.9^14^.

**Table 2:** Optimized parameters used for each analyzing step on the KBase platform.

Step	Software	Version	Parameters
Filter and Trim	FastQC	0.11.5	Default
	Trimmomatic	0.36	Adapters: TruSeq. 3-PE-2; Minimum read lenght: 70 bp; Minimum quality: 30
Contigs co-assembly	Merge Reads Libraries	1.0.1	Default
	MEGAHIT	1.2.9	Meta-large assembly; Minimum contig length: 2000 bp
Binning	MetaBat2	1.7	Minimum contig length: 2000 bp
	Maxbin2	2.2.4	Probability threshold: 0.8; Marker set: 107 and 40; Minimum contig length: 2000 bp
	Concoct	1.3.4	Read Mapping Tool: Bowtie2; Minimum contig length: 2000 bp
	DasTool	1.2	Gene Identification Tool: Blast
Quality Check	CheckM	1.0.18	Full reference tree
	Metagenome BinQC	1.1.2	Default
Coverage	BBMap	1.3.0	Default
Taxonomic Classification	GTDB-Tk	2.3.2	Default
Phylogenetic Analysis	Build Microbial Species Tree	1.6.0	Default

Since differences in binning methods or software may introduce biases^15^, we performed binning using contigs >2000 bp with a combination of three algorithms: MaxBin2 2.2.4^16^, MetaBAT2 1.7^17^, and CONCOCT 1.3.4^18^, followed by consensus binning with DAS Tool 1.2^19^ to improve bin quality and annotation accuracy. CheckM 1.0.18^20^ and Metagenome BinQC 1.1.2^9^ were used to determine the number of rRNAs and tRNAs, completeness and contamination. According to the criteria established by Bowers *et al*.^11^, bins are typically classified as high-quality (≥90% completeness, ≤5% contamination, presence of at least one copy of the 5S, 16S, and 23S rRNA genes, and ≥18 tRNAs), medium-quality (>50% completeness and <10% contamination), or low-quality (<50% completeness and/or >10% contamination) assemblies. In this study, we applied a more stringent threshold and considered as medium-quality only the bins with >70% completeness and <10% contamination. This approach was intended to minimize bias from low-quality bins and reduce the likelihood that unclassified bins resulted from poor assembly quality^21,22^. Bins meeting the high- and medium-quality thresholds were taxonomically classified using GTDB-Tk v2.3.2^23^. The percentage of mapped reads and coverage of the MAGs in relation to its co-assembly sequence libraries were estimated using BBMap 1.3.0^9^. The graphical visualization was carried out on R 4.0.5^24^ using the package ggplot2 3.5.1^25^. The Kruskal-Wallis test, followed by Bonferroni correction for multiple comparisons, was applied to compare the completeness, contamination, GC content, and genome length across the different treatments and depths, using the ggpubr package 0.6.0^26^. Lastly, MAGs were used as inputs for phylogenetic analysis based on a set of 49 universal core genes defined by Clusters of Orthologous Groups (COG) gene families by the app Build Microbial SpeciesTree 1.6.0^9^ and the phylogenetic tree was generated using TreeViewer^27^.

## Data Records

The 36 raw shotgun metagenome reads generated in this study are publicly available on the NCBI Sequence Reads Archive (SRA) under the BioProject PRJNA1160830^28^. The 160 reconstructed MAGs have been deposited in the GenBank database^29^ under accession numbers JBIOZP000000000-JBIOTM000000000, and their fasta files have also been made accessible through figshare^10^. Detailed information pertaining to all the reconstructed MAGs, including their corresponding BioSample and GenBank accession numbers, is detailed in **Quality Metrics File**^10^.

## Technical Validation

The quality of MAGs was assessed using CheckM to validate the contamination and the completeness of the bins. Additionally, the Metagenome BinQC was used to confirm the quality parameters besides genome statistics, that includes genome size, N50, L50, GC content, and the estimated number of rRNA and tRNAs genes.

## Data Availability

Custom scripts were not utilized for the generation and processing of this dataset. Software versions and any non-default parameters used have been explicitly specified on the Table 2.
